# The Strengths and Difficulties Questionnaire and standardized academic tests: Reliability across respondent type and age

**DOI:** 10.1371/journal.pone.0220193

**Published:** 2019-07-25

**Authors:** Maria Keilow, Hans Henrik Sievertsen, Janni Niclasen, Carsten Obel

**Affiliations:** 1 VIVE–The Danish Center for Social Science Research, Copenhagen, Denmark; 2 Department of Public Health, Aarhus University, Aarhus, Denmark; 3 Department of Economics, University of Bristol, Bristol, United Kingdom; 4 Frederikshøj skole og dagbehandling, Primulavej 2, DK-2720 Vanløse and Steno Diabetes Center Copenhagen, Capital Region of Denmark, Health Promotion, Diabetes Prevention Research, Gentofte, Denmark; University of Copenhagen, DENMARK

## Abstract

Exploiting nation-wide data from the Danish National Birth Cohort, we show that children’s emotional and behavioral problems measured by the Strengths and Difficulties Questionnaire (SDQ) are closely related to their performance in standardized academic tests for reading and mathematics in sixth grade. The relationship is remarkably linear across the entire distribution for both the total difficulties score and subscale scores of the SDQ; higher scores on the SDQ (more problems) are related to worse performance in academic tests. We assess the similarity across respondent type; parent (child age 7 and 11), teacher (child age 11) and self-reported scores (child age 11), and find that teacher and parent reported scores have very similar slopes in the SDQ–test score relationship, while the child reported SDQ in relation to the academic test performance has a flatter slope.

## Introduction

While individual behavior and mental health are interesting outcomes in themselves, the growing literature on the importance of these characteristics for educational achievement and labor market outcomes [1–4] has sparked the demand for validated instruments. One widely used potential candidate for measuring children’s emotional and behavioral problems is the Strengths and Difficulties Questionnaire (SDQ), which is increasingly used by economists, sociologists [5–14], and by other professionals.

The SDQ has been extensively evaluated for its internal consistency, test-retest reliability, and inter-rater agreement [1,15]. However, less is known about how the SDQ relates to measures of academic test performance across respondent type and child age.

The objective of this paper is twofold: First, we assess how the SDQ is related to performance in standardized academic tests across three respondent types (parent, teacher, and child self-reported scores) and two age-levels of the child (age 7 and 11). Second, we exploit our sample size to nonparametrically identify the functional form of the relationship between the SDQ and the performance in standardized academic tests, both on the aggregate level (total difficulties score) and for each of the five subscales of the SDQ (hyperactivity/inattention, conduct problems emotional symptoms, peer relationship problems, and prosocial behavior). In other words, this paper assesses whether the SDQ can be used as a continuous outcome, and whether it matters, if scores from the parent, teacher or child self-reported versions of the SDQ questionnaire are used.

The study most similar to ours is one carried out by Kristoffersen et al. [12], who show that teacher and parent reported SDQ is closely associated with academic performance in ninth grade final exams. Kristoffersen et al. also find that teachers are more likely than parents to report extreme SDQ values (very low/high level of problems). Compared to Kristoffersen et al. [12], we add the self-reported version of the SDQ for children aged 11 in our analyses. Furthermore, we assess the functional form of the relationship at the aggregate as well as the subscale level. The latter is important, as studies using SDQ data typically focus on differences across the three pre-defined thresholds; normal, borderline and abnormal, although variation within these categories might also be informative. We therefore assess whether the relationship between the SDQ scores and the performance in standardized academic tests reflects these three groups, or whether the variation within the groups is informative.

## Methods

### Data

Data for this study consists of registry data and data collected by the Danish National Birth Cohort (DNBC). The study was approved by the National Agency for Education and Quality under the Danish Ministry of Education, the Danish National Birth Cohort Steering Committee, and the Danish Data Protection Agency, respectively. Data were analyzed anonymously on secure servers at Statistics Denmark in line with current data protection requirements. Regional ethics committees approved the collection of data for the DNBC prior to this study. Consent was collected from parents and teachers for data being used for research purposes in accordance with the approved procedures.

### Measurements

#### The Strengths and Difficulties Questionnaire

The SDQ was developed by the English child psychiatrist Robert N. Goodman. The questionnaire assesses emotional and behavioral problems in children and adolescents [16–18]. It consists of 25 items and an impact part and has the advantage of being relatively short and uniform across respondent type (parent, teacher or child) [19]. In addition, the inclusion of positive items makes SDQ suitable across non-clinical samples. The established scoring procedure for the SDQ links each of the 25 items to one of five distinct subscales: emotional symptoms, conduct problems, hyperactivity/inattention, peer relationship problems, and pro-social behavior. The sum scores for each of these scales range from zero to ten. The first four categories can be aggregated by adding the subscale scores to a total difficulties score (ranging from zero to 40 points). In the nonparametric part of the analysis, we exclude SDQ levels with less than 100 observations due to issues of imprecision. Consequently, the graphs do not necessarily show the entire range of the scale. All observations are, however, included in the linear regression analysis.

#### The Danish National Birth Cohort (DNBC)

The DNBC is a longitudinal nationwide cohort and includes over 100,000 pregnancies (from more than 92,000 women) enrolled from 1996–2002, representing 31 percent of all pregnancies in Denmark during that period [20]. The DNBC cohorts thus include children, who were born from 1997–2003. The women were interviewed twice during pregnancy and when the child was 6 and 18 months, 7 and 11 years. In this paper, we use data from the fifth wave (children age 7) and the sixth wave (children age 11) of the DNBC. Both waves contain parent reported SDQ scores. The sixth wave also includes child and teacher reported SDQ scores.

#### The Danish National Tests

To measure student academic achievement, we include data from the Danish National Tests taken in the years 2010–2014. These academic tests are self-scoring and adaptive tests that are provided from the Danish Ministry of Education systematically and nationwide throughout compulsory public schools with certain subjects tested at certain grade levels [21]. We use tests in mathematics and reading in sixth grade, when the children are approx. twelve to thirteen years old. The tests at this grade level were chosen a priori to the study because of their timing in relation to the SDQ measurements for the available cohorts in the study sample; i.e. children take these tests at age 13 and thus the tests are subsequent to the SDQ measurements at age 7 and 11. Moreover, at sixth grade, tests are available for both mathematics and reading. In all analyses, academic test scores are standardized to a mean of zero and a unit standard deviation.

### Sample selection

We link data on SDQ scores from the DNBC [22] to data on results from the Danish National Tests [21] and a number of socioeconomic covariates. Of the 461,635 children born between 1997 and 2003 and observed in the register data, we can match 68,602 to at least one complete SDQ score in the DNBC and of those, we are able to match 42,863 children to sixth grade test scores in the Danish National Tests in either reading or mathematics. Last, 1095 children are excluded due to missing covariates. The final sample consists of 41,768 children, corresponding to nine percent of the relevant birth cohorts, i.e. the 1997–2003 cohorts.

There are three sources of non-random selection: First, selection bias due to non-participation may occur. Women were recruited to the DNBC through their general practitioner at routine health control visits during their pregnancy. Approx. two-thirds of those women, who were invited to participate in the DNBC, enrolled and completed at least one interview. Groups with lower socioeconomic resources in terms of occupation, education, and income are underrepresented in the DNBC compared to the general population [20,22,23]. Since participation in the DNBC required Danish language skills, families with non-western origin are also underrepresented. Teachers of children participating in DNBC were recruited for the age 11 follow-up and included approx. 19,000 teachers, corresponding to 65 percent of those, who were invited. Second, the Danish National Tests only cover children in public schools. Selection into private schools may be related to emotional or behavioral problems. In addition, children with severe mental health problems may be excused from taking the test. The relationship between the SDQ scores and the standardized academic test scores might therefore be different for the children not covered by our data. Third, we only include complete cases. This sample selection criterion is not very restrictive, as all covariates are observed for nearly all observations, i.e. for 97 percent.

### Statistical analyses

We first describe sample characteristics in terms of variable means. We compare population means to the study sample means and compare the study subsample means among each other to assess external and internal validities, respectively.

Next, we present nonparametric analyses of the relationship between SDQ scores (total difficulties score and subscale scores) and standardized academic test scores (reading and mathematics). So far, researchers have very often relied on the binary or categorical scoring of the SDQ in categories of abnormal/borderline vs. normal scores [12]. To the best of our knowledge, there is no evidence of whether variation in the SDQ scores away from these thresholds is correlated with school performance. To assess this, we compute mean academic test scores for each level of the total difficulties scale and present this relationship in graphs. We compare the relationship for all respondent types; parents (children age 7 and 11), teachers (children age 11) and children age 11.

Last, we regress academic test scores on the SDQ scores using ordinary least squares. We restrict data to complete case analyses. Estimating these linear regressions, we present unconditional estimates and estimates conditioned on a rich set of controls that include parental education, income, and age at childbirth, child gender, birthweight and non-western origin. We also include school fixed effects in the conditional models to correct for unobservable variation across schools. These regression results are presented for reading and mathematics test scores, for SDQ total difficulties score as well as subscale scores and for each respondent type. While parametric tests rely on assumptions regarding both distribution and functional form, semiparametric tests rely on one or the other (e.g., our linear regression results assume a linear functional form). The graphs presented rely on neither and are therefore considered nonparametric. All analyses are performed using Stata version 14.

## Results

### Sample characteristics

Linking the DNBC data to administrative records from Statistics Denmark, Table 1 presents variable means by subsamples for all covariates included in the analyses. Parent educational level is measured as years of schooling for the parent with the longest education. Parents’ income is presented as total gross household income in 1,000 Euro (2010 price level), and equivalized by dividing the total household gross income by the square root of the number of household members. Non-western origin indicates that either or both parents have a non-western origin.

**Table 1:** Variable means by subsamples.

		Subsamples			
	(1) Population	(2) Parent, Age 7	(3) Parent, Age 11	(4) Teacher, Age 11	(5) Child, Age 11
Female	0.49	0.50	0.52	0.52	0.54
Birthweight (gr.)	3,498	3,568	3,579	3,597	3,577
Non-western origin	0.11	0.01	0.01	0.01	0.01
Parents’ years of schooling	14.59	15.58	15.72	15.92	15.75
Gross household income (1,000 Euro)	40.69	45.86	46.31	46.83	46.26
Mother’s age at child birth	30.00	30.57	30.70	30.82	30.72
Father’s age at child birth	32.67	32.79	32.87	33.01	32.89
Observations	461,635	33,584	28,919	11,819	26,458

*Source*: Own calculations on data from Statistics Denmark and from the DNBC. Parental variables are measured in the calendar year before the child was born. Column (1) shows variable means for all children born 1997–2003. Columns (2) to (5) indicate respondent type subsample.

Table 1 shows that for the external validity of our results, children in our sample have higher birthweight, and their parents have higher incomes and have completed more years of schooling compared to the general population. In addition, families with non-western origin are underrepresented in the sample. Statistical t-tests show that these differences are significant. However, important for the internal validity of our study, the subsamples in columns (2) to (5) have remarkably similar means. While the selection into the sample is non-random, as suggested, given at least one SDQ score is observed, there is no selection across subsamples of respondent type.

### SDQ and standardized academic tests

**Fig 1:**
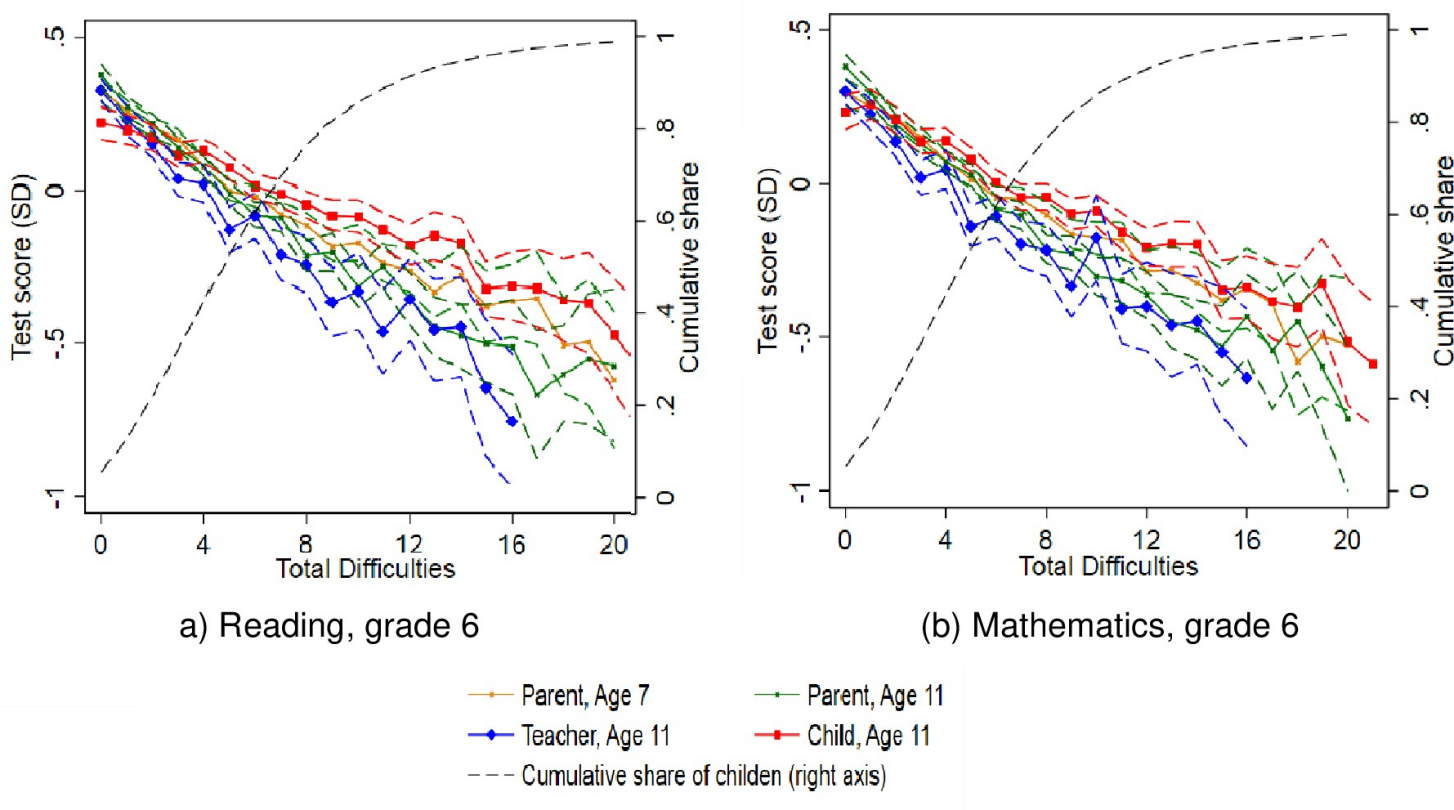
Relationship between SDQ total difficulties and academic test scores in reading and mathematics in sixth grade. *Source*: Own calculations on data from Statistics Denmark and from the DNBC. Mean academic test scores for each level of the total difficulties scale. Dashed lines indicate pointwise 95 percent confidence bands. The graphs only contain SDQ levels with at least 100 observations, corresponding to at least 98 percent of the sample. The cumulative share of children observed (shown on the right axis) refers to the parent-reported SDQ scores at age 7.

We now turn to the relationship between SDQ scores and children’s performance on the standardized academic tests. We focus on tests in reading and mathematics in sixth grade. Fig 1 presents the relationship between children’s SDQ total difficulties scores and their standardized academic test scores.

Fig 1 shows the plots for reading and mathematics test scores, respectively. In these graphs, we exclude total difficulties levels with fewer than 100 observations, as these estimates become very imprecise. (All observations are, however, included in the regressions presented in the following). The dashed line shows the cumulative share of children observed; from this line, we see that 80 percent of the observations have total difficulties scores of nine or less, and 98 percent of the observations have scores of 21 or less.

We first note that for both subjects (reading and mathematics) and all respondent types, the relationships are remarkably linear. Thus, the relationship between total difficulties and standardized academic test scores is very constant, independent of whether we are looking at variation at the lower end of the distribution (few problems) or further to the right in the distribution of the total difficulties score. We also note, however, that some differences appear across respondent types as the total difficulties score increases: The steepest (flattest) slopes are observed for the teacher (child) reported scores, but overall the slopes are very similar across respondent type and child age. These observations are similar for reading and mathematics test scores.

**Fig 2:**
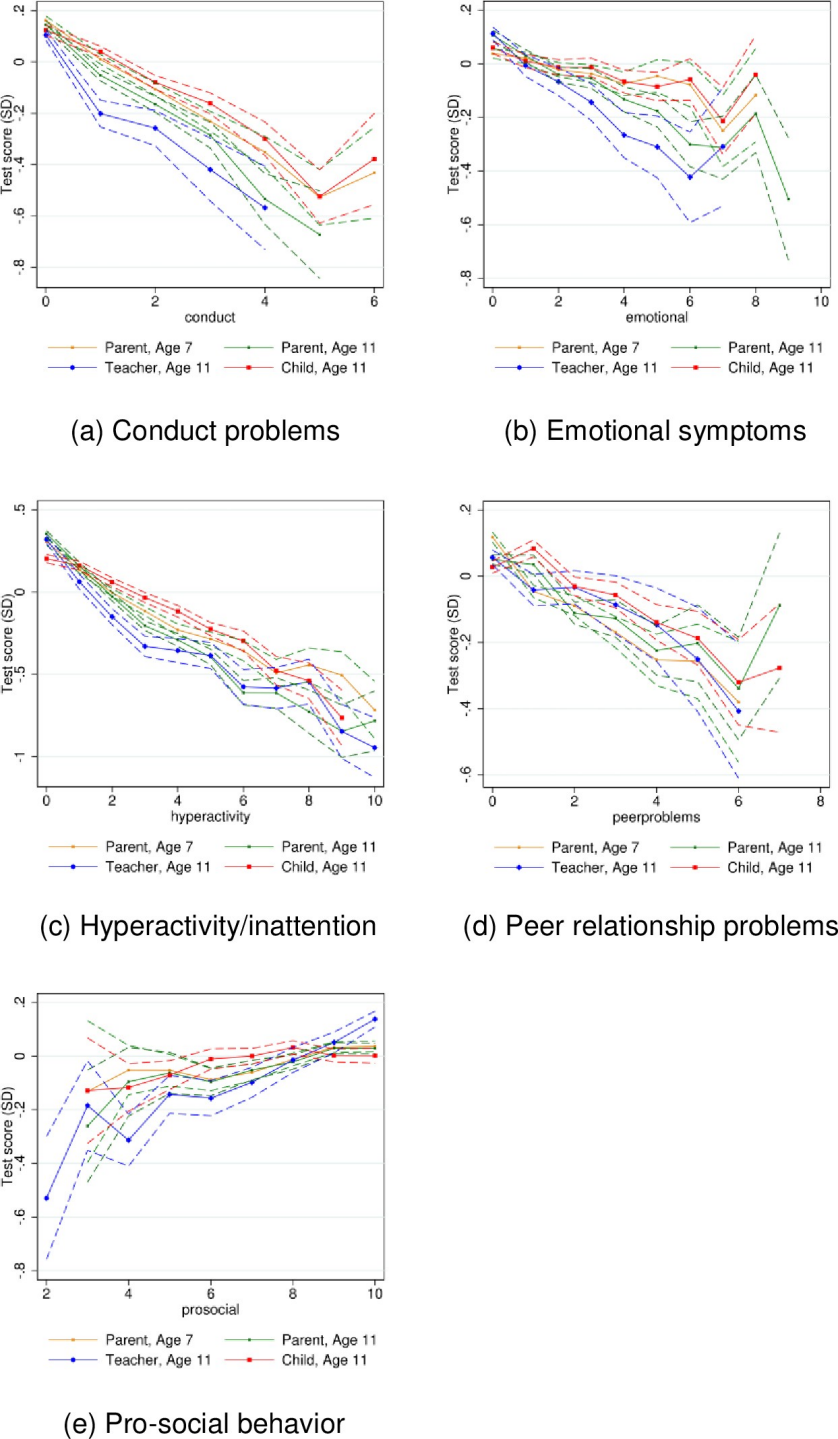
Relationship between SDQ subscales and reading test scores in sixth grade. *Source*: Own calculations on data from Statistics Denmark and from the DNBC. Mean reading test scores for each level of the SDQ subscale. Dashed lines indicate pointwise 95 percent confidence bands. The graphs only contain SDQ levels with at least 100 observations.

**Fig 3:**
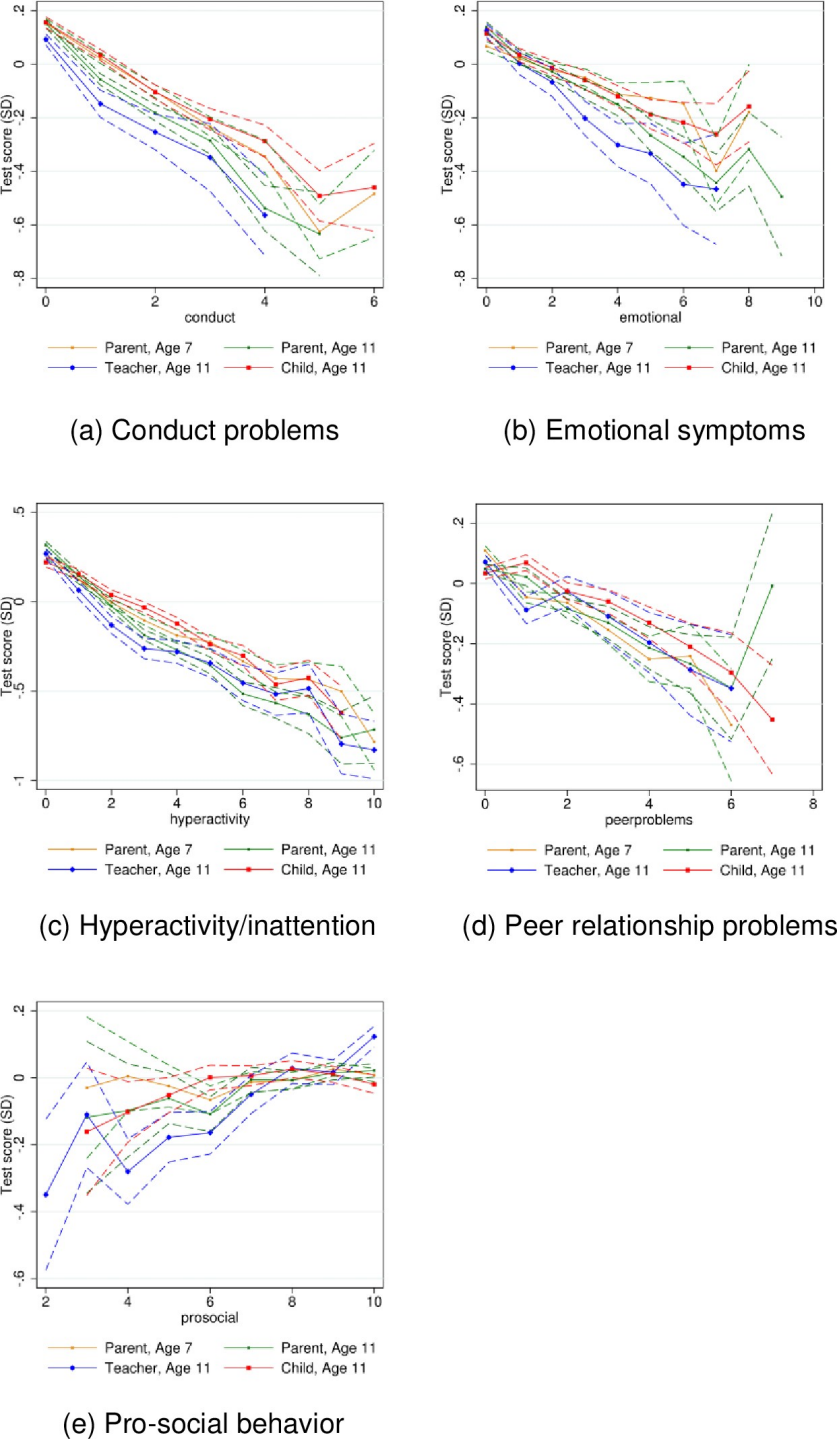
Relationship between SDQ subscales and mathematics test scores in sixth grade. *Source*: Own calculations on data from Statistics Denmark and from the DNBC. Mean mathematics test scores for each level of the SDQ subscale. Dashed lines indicate pointwise 95 percent confidence bands. The graphs only contain SDQ levels with at least 100 observations.

Fig 2 and Fig 3 show the nonparametric relationships for each of the five SDQ subscales for reading and mathematics test scores, respectively.

From Fig 2 and Fig 3 we see that especially the scales for hyperactivity/inattention and conduct problems show very tight and linear relationships between levels of difficulties and academic test performance, across all respondent types. Again, although the slopes are very similar across respondent type and child age, the flattest slopes are observed for the child reported SDQ.

As the nonparametric analyses clearly indicate a linear relationship between the SDQ and the academic test scores, including the SDQ subscale scores, a linear model is warranted. Table 2 presents result from estimating a linear model regressing academic test scores on the SDQ total difficulties scores. We present unconditional estimates and estimates conditioned on a rich set of controls using ordinary least squares. (All control variables included in the analyses are presented in Table 1).

**Table 2:** Regression results for SDQ total difficulties. Dependent variable: Academic test score in reading (A) and mathematics (B) in sixth grade.

	(1) Parent, Age 7	(2) Parent, Age 11	(3) Teacher, Age 11	(4) Child, Age 11
*A*. *Reading*				
Total difficulties, unconditional	-0.041** (0.001)	-0.050** (0.001)	-0.050** (0.002)	-0.030** (0.001)
Total difficulties, conditional	-0.027** (0.001)	-0.038** (0.001)	-0.042** (0.002)	-0.021** (0.001)
Observations	33,167	28,563	11,598	26,142
*B*. *Mathematics*				
Total difficulties, unconditional	-0.043** (0.001)	-0.053** (0.001)	-0.051** (0.002)	-0.036** (0.001)
Total difficulties, conditional	-0.032** (0.001)	-0.042** (0.001)	-0.046** (0.002)	-0.027** (0.001)
Observations	33,079	28,483	11,550	26,087

*Source*: Own calculations on data from Statistics Denmark and from the DNBC.

* p < 0.1

** p < 0.05.

Standard errors are clustered on the school level. Point estimates from separate regressions. In Panel A (B), the dependent variable is the sixth grade reading (mathematics) test score. Academic test scores are standardized to a mean of zero and a unit standard deviation. The first row in each panel shows point estimates from regressions that only include a constant and the total difficulties score. The second row shows point estimates from regressions where we also control for school fixed effects, parental education, parental income, parental age at child birth, child gender, child birthweight and non-western origin. Columns (1) to (4) indicate respondent types for the included SDQ score.

Panel A, Table 2 shows point estimates for reading for unconditional regressions (without controls) and for conditional regressions, where we control for school fixed effects, parental education, parental gross income, parental age at child birth, child birthweight, gender, and non-western origin. One additional unit on the total difficulties scale (indicating more difficulties) is associated with three to five percent of a standard deviation lower academic test score, unconditionally, and two to four percent of a standard deviation lower test score, conditionally.

For mathematics (Panel B, Table 2), the coefficients are slightly larger; three to five percent of a standard deviation lower test score, conditionally. Teacher and parent reported SDQ have remarkably similar coefficients for same-aged children (age 11). In line with the presented graphical evidence, the estimates for child reported SDQ scores have the lowest coefficients of the three respondent types.

Table 3 shows regression results by the SDQ subscales conditioned on the control variables.

**Table 3:** Regression results for each SDQ subscale. Dependent variable: Academic test score in reading (A) and mathematics (B) in sixth grade.

	(1) Parent, Age 7	(2) Parent, Age 11	(3) Teacher, Age 11	(4) Child, Age 11
*A*. *Reading*				
Conduct problems	-0.079** (0.004)	-0.102** (0.005)	-0.120** (0.008)	-0.068** (0.004)
Emotional symptoms	-0.002 (0.003)	-0.034** (0.003)	-0.063** (0.005)	-0.018** (0.003)
Hyperactivity/inattention	-0.075** (0.003)	-0.111** (0.003)	-0.111** (0.004)	-0.064** (0.003)
Peer relationship problems	-0.039** (0.005)	-0.024** (0.004)	-0.027** (0.006)	-0.020** (0.004)
Pro-social behavior	0.013** (0.003)	0.013** (0.004)	0.037** (0.004)	-0.004 (0.003)
*B*. *Mathematics*				
Conduct problems	-0.090** (0.004)	-0.116** (0.005)	-0.132** (0.009)	-0.086** (0.004)
Emotional symptoms	-0.022** (0.003)	-0.050** (0.003)	-0.073** (0.005)	-0.035** (0.003)
Hyperactivity/inattention	-0.077** (0.003)	-0.110** (0.003)	-0.116** (0.004)	-0.071** (0.003)
Peer relationship problems	-0.047** (0.005)	-0.030** (0.005)	-0.039** (0.006)	-0.025** (0.004)
Pro-social behavior	0.013** (0.003)	0.019** (0.004)	0.047** (0.005)	0.002 (0.004)

*Source*: Own calculations on data from Statistics Denmark and from the DNBC.

* *p* < 0.1

** *p* < 0.05.

Standard errors are clustered on the school level. Point estimates for separate regressions. In Panel A (B), the dependent variable is the sixth grade reading (mathematics) test score. Academic test scores are standardized to a mean of zero and a unit standard deviation. All regressions include school fixed effects and controls for parental education, parental income, parental age at child birth, child gender, birthweight and non-western origin. Columns (1) to (4) indicate respondent types for the included SDQ score.

In line with the graphical evidence (Fig 2 and Fig 3), the relationship between SDQ scores and academic test performance presented in the regression estimates (Table 3) is strongest for conduct problems and hyperactivity/attention, whereas the relationship for prosocial behavior seems the weakest.

The results for the parent reported SDQ scores indicate that the association with academic test performance is stronger at age 11 than at age 7. For example, whereas one additional unit higher score for hyperactivity/inattention is associated with eight percent of a standard deviation lower test scores at age 7, it is associated with eleven percent of a standard deviation lower test scores at age 11. This tendency is visible for all subscales except peer relationship problems.

## Discussion

Using data from Statistics Denmark and a largescale nation-wide birth cohort, we show that emotional and behavioral problems as measured by the SDQ are closely related to student performance in standardized academic tests of reading and mathematics in sixth grade. The relationship is remarkably linear across the entire distribution and for both the total difficulties scores and individual subscales.

While teacher and parent reported scores show very similar results, the relationship between the child’s self-reported SDQ and the academic test performance has a flatter slope. Normative data from various samples show that children report more extreme SDQ scores compared to both teacher and parent assessments for total difficulties as well as subscales [24,25]. If children report more emotional and behavioral difficulties (and more pro-social strengths), this difference in itself would parallel shift this graph to the right compared to the graph for teacher and parent respondents, though it would not necessarily translate into the flatter slope that we observe. However, this difference between children’s self-reported scores and parent or teacher scores is not stable across the distribution, rather the difference increases with higher self-reported scores. For example, our data shows that for those children who report a total difficulties score on the SDQ higher than the median 76 percent of parents report a lower score than their child does, indicating that they assess fewer problems than the child does. In fact, parents in this group on average report a four point lower total difficulties scores than the child self-report. For children who report scores lower than or equal to the median the difference is much smaller; here, parents on average report a 0.2 points higher score than the child and only 40 percent of parents provide lower scores than their child. It appears that for higher self-reported scores (more problems), parents are often more positive than their child and the difference in the assessment of problems quite large, whereas the assessments are much more similar at the lower end of the distribution. We believe that these differences in assessment at the higher end of the distribution explain the differences in the slopes that we observe.

Although the SDQ has been extensively examined for its internal consistency, test-retest reliability, and inter-rater agreement, less is known about how the questionnaire relates to academic performance outcomes such as standardized tests in school. Studies examining this relationship across respondent type and child age are even scarcer. This study adds to previous research by identifying the functional form of this relationship for three types of respondents and two age levels. We show that the variation in the SDQ scores away from the abnormal/borderline vs. normal thresholds (i.e. using SDQ as a continuous variable) is correlated with children’s performance in standardized academic tests. The results are remarkably similar for all types of respondents and age levels. To the best of our knowledge, this characteristic of the relationship between the SDQ and standardized academic tests has not been addressed in the literature.

### Strengths and limitations

A major strength of this study is the large sample size, which enable the nonparametric analyses of the functional form of the relationship between SDQ and academic test scores. In addition, using data from three respondent types and two age levels enables the comparison across these subsamples, which adds to the level of detail presented in the study.

Limitations of this study include the fact that the study sample consist of a non-random subsample of the population of children born from 1997–2003. The results are therefore not informative about the relationship between emotional and behavioral problems and academic test scores for children excluded from the DNBC due to non-response. Similarly, for children who have not completed the Danish National Tests (including children who do not attend a public school or children exempt from taking the test due to severe mental health problems or learning disabilities), we can only speculate about the relationship between their SDQ scores and academic performance.

### Implications

The SDQ is a reliable, valid and widely used instrument for identifying emotional and behavioral problems in primary school children in research and among professionals. Assessing the functional form on both aggregate and subscale levels of the SDQ in terms of academic test scores, this study provides relevant information on the reliability and use of the questionnaire. Due to limited knowledge about the informative value of the lower end of the SDQ distribution (fewer problems), researchers have so far have been reluctant to use the raw subscale scores. The results of this study imply that the use of SDQ scores as continuous variables is justified and that the lower end of the SDQ scale is informative, even at a subscale level.
